# Some General Considerations

**Published:** 1908-09-05

**Authors:** 


### Post-Graduate Medical Study.

### SOME GENERAL CONSIDERATIONS.

"He who wishes to be a good physician must be a student all his life long. Only by constant and unfailing observation and study can he attain to expertness," declared the great Boerhaave, *facile princeps* among the doctors of his day. No man, it is safe to say, carried principles into practice with so much courage as did the Leyden physician. He remained a student to the close of his life. One has only to glance at his letters—at those crabbed, nearly undecipherable Latin epistles of which the Sloane collection contains a few good specimens, to perceive how keen he was to learn. "I thank you for your information," he writes. "It is new to me, and I must think over it." That, in fine, is the kernel of post-graduate study—to see something new, and to think it over, accepting it if it is found good, and unhesitatingly rejecting it if it is found bad. Graduate study is not a new departure. When he had finished his academic education—"become qualified" as we would nowadays say—Galen undertook a three years' trip to the large centres of medical education in Egypt and Palestine, to complete his survey. Similarly Baglivius, several hundred years later, came to England for graduate study purposes; the younger Moore travelled extensively, and if one pleased one could add many other examples to the list. But there is this difference between graduate students of the past and those of to-day, namely, that then it was the specialist, the professor, the leader who went abroad, or took special courses. The general practitioner had no time, no opportunity, and no means, even if he had the desire, to become a graduate student. He lived in his little world, contented with his lot, or grumbling and swearing, in that delightfully diabolical way which Dr. Messenger Monsey cultivated, at "the mischance that made him a plaster on the back of a lousy Devonshire village." Nowadays, however, post-graduate study offers many attractions to the ordinary practitioner. Even the man who is engaged in a busy general practice will find opportunities and facilities for "brushing up" his knowledge in the various departments of medicine. He owes it to his patients, nay more, he owes it to himself, to take advantage of such opportunities, and to do his utmost to keep abreast of the advances in modern science and their special bearing upon the branches in which he is interested.

There are many ways in which the graduate student can set to work. He may do independent private study. That is to say, he may obtain the latest text-books and literature on the subjects in which he takes an interest, and study these as he used to do in his hospital days. This method is laborious, and it demands an outlay of time and trouble which is, when all things are considered, hardly commensurate with the benefits it offers. A vast amount of time and energy is necessary to abstract from current literature and the latest text-books just what is wanted. In general the practitioner will find it advantageous to read one or two medical journals, and to pay particular attention to such abstracts of current advances which are worth noting, as may from time to time be published in their columns.

A second way in which to study is to take advantage of one or other of the various facilities which are nowadays offered to the practising physician to brush up his knowledge. What these facilities are we need not here stop to describe. They have been fully explained from time to time in the columns of The Hospital, and in the present issue there will be found a full summary of the various institutions that offer advantages to the graduate student. Let us say at once that all of them are worth the earnest attention of the practitioner. They do not all work on identical lines, and therefore one or other will be more suitable for the practitioner who finds himself completely in harmony with its lines, while another, again, will attract the man who finds its particular merits carry away his full satisfaction. The main thing is that there are such institutions, and that the practitioner should make use of them. They have this great advantage over private study, that they are selective, and economical. The student is offered compressed knowledge; it is graduate study in tabloid form, using the word tabloid in no derogatory sense! The teachers are men who understand just what the student wants, and try to give it him. From all that we have seen and heard of these institutions, we can cordially recommend them. They are in every way graduate institutions, catering for graduate men only, and offering to practitioners the fullest advantages, and the most admirable facilities for graduate study.

It may not be out of place to say a few words here on the important subject of how graduate study should be commenced. We have in mind a genial practitioner, common-sense to the core, but with just a trifle too much of human fulminate in his composition, who attended a pathology class at a graduate study institution. This gentleman, who had qualified in the early eighties, had the laudable desire to brush up his knowledge, and when a week after, he had signified his intention of attending X———'s class at the ———, we met him, we were surprised to find that he had nothing good to say of the course. "Would you believe it," he declared, "the chap said I ought to have a new microscope. I've had my binocular for twenty years, and I'll not buy a new microscope just to see a spirochete with!" This, we need scarcely say, is not the attitude which the true graduate student should adopt. For all that it is one which many, unconsciously reveal when they first participate in a course. The mildly deprecating manner of the demonstrator, who points out that instruments and methods in use twenty years ago are hardly suitable for present-day work, may be irritating. We have found it so ourselves on occasions, but the real graduate student should be far above taking offence at manner or word. He should carefully consider, before he puts down his name for a course, what his membership means. Obviously he cannot expect to work equally well with antiquated instruments as with up-to-date ones such as his colleagues are equipped with. A preliminary letter to, or verbal explanation with the demonstrator or the colleague who gives the course, will usually clear up all difficulties, and in the end the student will find that the purchase of the necessary new instruments has not been unnecessary expense, but that he can use his novelties in his daily work. In fact, in order to practise the methods that he learns in his course, he requires new and up-to-date apparatus, and it is much the most sensible thing to purchase these when he starts a course, and thus to familiarise himself with their working. This is also true of new text-books. A variety of these the student will find indispensable. His library, even if he specialises very strongly, will have to be catholic to some degree. Specialities overlap even now, and the tendency towards greater overlapping is every day becoming stronger.

A third method is to travel abroad, and to work as a free lance or to "take courses" at some hospital or university where the methods are radically different from those the student already knows. This is perhaps the ideal method; it is certainly the one which combines pleasure and profit in a manner which is highly satisfactory. Unfortunately it is not one that can be used by every practitioner. The annual holiday is too short to be used as a period of study, and during his active practice the student graduate has no opportunity for travelling. Nevertheless the annual holiday may be used to a certain extent. The practitioner will find opportunities for study even when he is spending his vacation cloistered in some quiet country nook. When he goes abroad there are many opportunities. He may run round the hospitals, always sure of a warm welcome from his colleagues, even if the means of expressing it, owing to mutual ignorance of each other's language, is not verbally strong! By such chance visits to foreign hospitals he will learn much if he uses his powers of observation. Still more will he learn if he attaches himself for a short time to one or other of the better-known teachers. To do so he need not necessarily always take a course. A personal introduction is all that is necessary in most cases. Going round with such a distinguished colleague, he will find, is worth a great deal. New methods are to be observed, new methods in diagnosis, in treatment, and in examination. These he will have to compare and consider, and that in fact is the essential point in graduate study. It is rare for the man who has learnt his work well to find something that is absolutely new to him when he goes round as a graduate student. What he finds is a new application of knowledge that he already possesses. He learns to criticise, to differentiate, and to compare—and how valuable all that is for the medical man whose daily professional life is a round of criticism, comparison, and differentiation!

Personally we know of few pleasures, in a professional sense, that can compare with this contemplation of the methods and work of our colleagues abroad. There is not only the attraction of visiting new places, of seeing new sights, of finding new interests in life, but there is the added attraction of being able to make all this of real use to us in our daily work. Such "study travel" makes a full man professionally speaking, much more than does the mere constant reading of text-books and current literature. Of its other pleasures one might be inclined to write a page. The colleagues one learns to regard as friends, while the recollection of the time spent at this or that "centre," working with this or that "master" remains one of indelible pleasure to the graduate student in after days.

